# Adiabatic Invariant of Center-of-Mass Motion during Walking as a Dynamical Stability Constraint on Stride Interval Variability and Predictability

**DOI:** 10.3390/biology11091334

**Published:** 2022-09-09

**Authors:** Fabien Buisseret, Victor Dehouck, Nicolas Boulanger, Guillaume Henry, Florence Piccinin, Olivier White, Frédéric Dierick

**Affiliations:** 1 CeREF-Technique, Chaussée de Binche 159, 7000 Mons, Belgium; 2 Forme and Fonctionnement Humain Laboratory, Department of Physical Therapy, Haute Ecole Louvain en Hainaut, rue Trieu Kaisin 136, 6061 Montignies-sur-Sambre, Belgium; 3 Service de Physique Nucléaire et Subnucléaire, UMONS Research Institute for Complex Systems, Université de Mons, 20 Place du Parc, 7000 Mons, Belgium; 4 Service de Physique de l’Univers, Champs et Gravitation, UMONS Research Institute for Complex Systems, Université de Mons, 20 Place du Parc, 7000 Mons, Belgium; 5 Cognition, Action et Plasticité Sensorimotrice (CAPS), INSERM UMR1093, UFR STAPS, Université de Bourgogne Franche-Comté, BP 27877, 21078 Dijon, France; 6 Laboratoire d’Analyse du Mouvement et de la Posture (LAMP), Centre National de Rééducation Fonctionnelle et de Réadaptation—Rehazenter, Rue André Vésale 1, 2674 Luxembourg, Luxembourg; 7 Faculté des Sciences de la Motricité, UCLouvain, Place Pierre de Coubertin 2, 1348 Louvain-la-Neuve, Belgium

**Keywords:** gait variability, adiabatic invariant, random noise, metronome, Hurst exponent, phase space

## Abstract

**Simple Summary:**

Human walking exhibits properties of both stability and variability. On the one hand, the variability of the interval of time between heel strikes is autocorrelated, i.e., not randomly organized. On the other hand, walking is highly stereotyped and arguments from general mechanics suggest that the stability of gait can be assessed according to invariant properties. This study aims at proposing one of those invariants. Participants walked for 10 min at a natural pace, with and without a metronome indicating participants’ preferred step frequency. In both cases, we use different parameters to assess both the variability and stability of walking. We verify a known result: the metronome strongly alters the variability of the motion. However, despite the large variability changes, our proposed adiabatic invariant is preserved in both conditions, demonstrating the stability of gait. It appears as though our model reveals dynamical constraints that are “hidden” beyond apparent walking variability.

**Abstract:**

Human walking exhibits properties of global stability, and local dynamic variability, predictability, and complexity. Global stability is typically assessed by quantifying the whole-body center-of-mass motion while local dynamic variability, predictability, and complexity are assessed using the stride interval. Recent arguments from general mechanics suggest that the global stability of gait can be assessed with adiabatic invariants, i.e., quantities that remain approximately constant, even under slow external changes. Twenty-five young healthy participants walked for 10 min at a comfortable pace, with and without a metronome indicating preferred step frequency. Stride interval variability was assessed by computing the coefficient of variation, predictability using the Hurst exponent, and complexity via the fractal dimension and sample entropy. Global stability of gait was assessed using the adiabatic invariant computed from averaged kinetic energy value related to whole-body center-of-mass vertical displacement. We show that the metronome alters the stride interval variability and predictability, from autocorrelated dynamics to almost random dynamics. However, despite these large local variability and predictability changes, the adiabatic invariant is preserved in both conditions, showing the global stability of gait. Thus, the adiabatic invariant theory reveals dynamical global stability constraints that are “hidden” behind apparent local walking variability and predictability.

## 1. Introduction

Human gait can be considered a quasiperiodic phenomenon. Despite the apparent stability of walking at a constant speed, gait exhibits an inherent local variability that can be observed at the level of basic parameters such as the stride-to-stride interval time, or simply referred to as the stride interval (SI). The global stability during gait is typically assessed by quantifying the whole-body center-of-mass (COM) motion. Local variability can be modulated to maintain global stability [1].

It is well known that gait variability is a hallmark of healthy individuals and has properties that are far more complex than stochastic variability. Since the seminal works of Haussdorff et al. [2,3], it has been shown that the variability, more exactly the predictability, of SI over a long period of time—typically several hundred gait cycles—is autocorrelated, just as it is in chaotic systems. These autocorrelations can be assessed by computing the Hurst exponent or H [4,5], which typically ranges from 0.7 to 0.9 in young healthy adults [6]. Note that H = 0.5 implies random variability. Although the physiological mechanism that generates SI autocorrelations is still controversial, neurodegenerative diseases significantly alter SI variability [7], and the use of indices other than H can help distinguish between different pathologies [8,9]. Other disturbances during walking, such as the execution of a cognitive task [10] or following a rhythmic auditory cue [11], also alter SI variability.

Realistic values for H can be obtained by resorting to simple mechanical models of the inverted pendulum type, in which one or two parameters are randomly updated at the beginning of each step [12,13]. Although mechanical approaches are used to model gait variability, it is worth noting that developments in Mechanics, such as action-angle variables in the Hamiltonian formalism, have proven to be remarkably successful in finding conserved quantities for complex and even chaotic systems [14,15], i.e., quantities with invariant value over time. Identifying such a conserved quantity for human gait would be to find dynamical constraints “hiding” behind SI variability that could provide new insights into how dynamical systems, in which behaviors evolve over time, maintain their current state or stability while allowing for variability/predictability. Whereas energy is not necessarily conserved over time with stochastic variation in system parameters, adiabatic invariants are good candidates for (almost) conserved quantities. An adiabatic invariant, *I*, is a quantity that remains approximately constant during the evolution of a dynamical system even under slow external changes, i.e., an adiabatic transformation [14]. One way to define an adiabatic invariant, I, relevant to the analysis of rhythmic human motion for a given degree of freedom Q(t) for which the kinetic energy has the standard form $E_{k}=\frac{m}{2}{\dot{Q}}^{2}$ [16], with *m* a mass scale, is through

(1) $$\bar{E_{k}}=\pi \;I\;f\;,$$

where π=3.1415…, *f* is a given cycle frequency (the inverse of its duration) and $\bar{E_{k}}$ is the averaged kinetic energy on the cycle under consideration. In the model (Equation (1)), $\bar{E_{k}}$ and *f* are assumed to change significantly over time, but their ratio, which is proportional to *I*, should remain invariant. Here, $\bar{E_{k}}$ during each gait cycle will be computed from the whole-body COM vertical displacement. The term “adiabatic invariant” will therefore refer to the quantity (1) computed from the vertical displacement of the COM. It is an important restriction, since in principle, adiabatic invariants may be found in the other directions and for other degrees of freedom while walking. Another important remark is the following. The term “invariant” will be used to denote a function of the dynamical variables whose value does not change over time during the evolution of a dynamical system, while the word “constant” will be used for a parameter whose value is not modified by the experimental condition. A textbook example is that of a simple pendulum without friction: Total energy is invariant but not constant, since it depends on the amplitude.

Biological systems are the most noteworthy nonconservative systems that derive forces from internal energy reservoirs [17]. In previous work, the adiabatic invariant has been successfully applied to human movement [17]. In biological systems, it is also important to note that adiabatic invariants can be considered only as an approximation and are not rigorously but rather approximately invariant.

Previous work has shown that the relations (1) holds for rhythmic arm movements [17,18] and for walking [19]. In the latter work, participants walked for 3 min, and SI variability was not examined. More, limit cycle attractors in phase space $\left(Q,\dot{Q}\right)$, i.e., the average gait cycle of a participant, were not drawn while they can also be used as a visual representation of gait pattern. Human motion, and particularly walking, is indeed highly stereotyped, though noisy, and gait patterns are highly consistent for an individual over time [20]. The attractors may also distinguish between walking and running, as the transition from walking to running can be viewed as a change from one stable attractor to another [21]. Therefore, these attractors could help distinguish between participants and conditions. The area enclosed by the phase-space trajectory of system over one cycle is the adiabatic invariant *I*.

From the perspective of understanding the role played by the neuro-musculoskeletal system in constraining coordination and reducing the degrees of freedom [22], adiabatic invariants of motion are relevant quantities, since two or more variables are linked by a single invariant. Therefore, the primary objective of this study is to investigate whether the adiabatic invariant model (1) holds in a population of healthy young adults walking at spontaneous speed on a motorized treadmill during a sufficient number (typically more than 500) of cycles to also assess SI variability, predictability, and complexity. The secondary objective is to understand how a constraint applied on the system, in our case, a rhythmic auditory cue (metronome) indicating preferred step frequency, impact global stability (assessed using the adiabatic invariant *I*) and SI variables. Our hypotheses were that Equation (1) holds during walking on a treadmill, regardless of the metronome constraint, and that the latter constraint exhibits significant variability and predictability indices, as shown in [11].

## 2. Materials and Methods

### 2.1. Protocol

The protocol was performed in a session of approximately 60 min. It was validated by the Academic Ethical Committee Brussels Alliance for Research and Higher Education (protocol B200-2021-123). Participants were healthy students recruited in the Department of Physiotherapy of the Haute Ecole Louvain en Hainaut (Montignies-sur-Sambre, Belgium). After being informed of the study, each participant signed an informed consent. The same experimenters (F.P. and G.H.) were responsible for the measurements.

First, participants’ age and biometric data (mass and height) were collected. Participants were asked to wear a tight outfit. Participants’ pelvic movements were recorded using a Vicon opto-electronic system (Vicon Motion Systems Ltd, Oxford Metrics, Oxford, UK) composed of 8 cameras (Vero v.2.2) with a sampling frequency of 120 Hz. Four 14 mm diameter reflective markers were placed on the pelvis of the participants following the Plug-In-Gait model (Oxford Metrics, Oxford, UK): Left Anterior Superior Iliac Spine (LASI), Right Anterior Superior Iliac Spine (RASI), Left Posterior Superior Iliac Spine (LPSI), and Left Posterior Superior Iliac Spine (RPSI).

Then, participants took place on the belt of an instrumented motorized treadmill (N-Mill, Motekforce Link, The Netherlands). The vertical ground reaction force and center of pressure of each foot was recorded at a sampling rate of 500 Hz using the manufacturer’s software (CueFors 2, Motekforce Link, The Netherlands). Spontaneous walking speed was determined and recorded during a 3-minute habituation period. After a 1-minute rest, the participant walked at a spontaneous speed on the treadmill for 10 min (control condition, CTRL). The average step frequency, *f*, was automatically computed by the CueFors 2 software. The positions of the 4 markers, ${\vec{x}}_{a}$, were recorded with the Vicon system via the Vicon Nexus software (v.2.7.1, Oxford Metrics, Oxford, UK). After a short break of 3 min, the participant walked on the treadmill at a spontaneous speed for 10 min with instructions to synchronize his or her steps with the clicks of a metronome whose tempo corresponded to the number of steps per minute calculated in the CTRL condition (metronome condition, METRO). This allowed the participants to adjust their gait tempo on a step basis whilst ensuring they adopted their own comfortable pace. The duration of the successive gait cycles and the positions of the 4 markers were recorded in the METRO condition.

**Figure 1:**
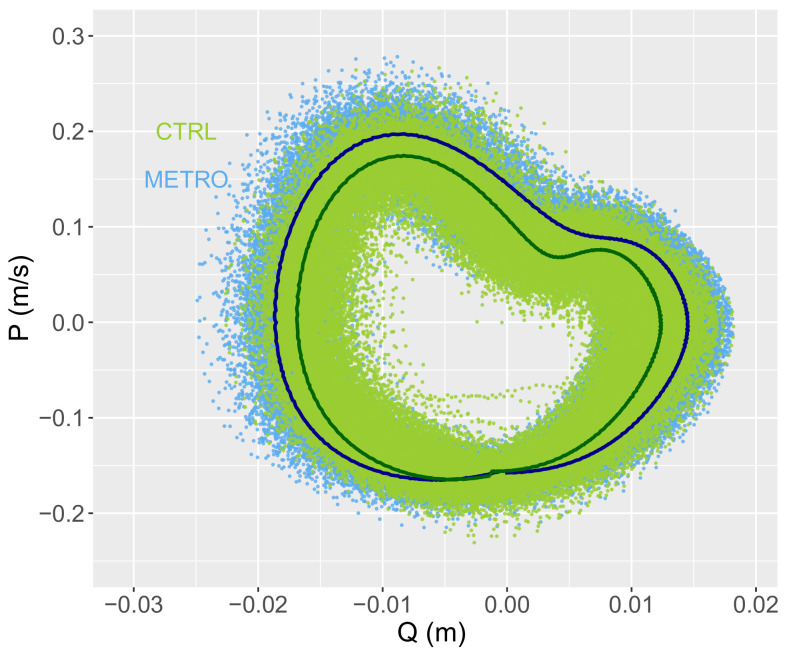
Two typical plots of whole-body COM vertical trajectories in phase space (Q,P) in the two studied conditions: CTRL (green) and METRO (blue). Attractors, computed as the mean cycle in phase space, are also displayed in the two studied conditions: CTRL (dark green line) and METRO (dark blue line). The same subject has been chosen in both conditions.

To estimate the whole-body COM vertical trajectory, Q(t), the mean vertical position of all pelvic markers (RASI, LASI, LPSI, and RPSI) was taken at each time step. Furthermore, to reduce measurement artefacts, Q(t) was filtered using a low-pass fourth-order Butterworth filter adjusted to each time series so that it kept 99.99% of the signal’s power. In order to alleviate the low sampling rate of the measurement system, a cubic spline interpolation was conducted on the data, multiplying the frequency by 10 to 1200 Hz. The speed and acceleration were then obtained through a finite difference scheme. The data was processed using R software (v. 4.1.0) [23]. Typical phase-space trajectories are shown in Figure 1.

### 2.2. Adiabatic Invariant *I* (Global Stability)

We assume that the vertical motion of the whole-body COM during walking is governed by the Hamiltonian $H=\frac{1}{2}\left(P^{2}+Q^{2}\right)\left(\omega +\epsilon \;\xi \left(t\right)\right)$, with 0<ϵ≪1 and ξ(t) a stochastic noise. This model is based on a forced harmonic oscillator with a frequency that fluctuates randomly around a mean ω. The noise accounts for physiological noise (i.e., the impossibility for the human locomotor system to be steadily in the same state) and should be low in healthy individuals. The momentum *P* is defined as $P=\dot{Q}$, as in standard Hamiltonian mechanics. This general class of Hamiltonians fits the form considered in the Appendix A, where the computational details are given. As developed in this Appendix A, the action variable (1) is an adiabatic invariant of the system: its value does not change over time except for small random fluctuations around the mean. In summary, the linear relationship (1) is to be expected in the analysis of the vertical motion of COM in healthy walking individuals.

A step is defined by the collection of data points between two maxima in Q(t). A gait cycle consists of two consecutive steps. For each gait cycle identified, denoted $C_{i}$, the frequencies $f_{i}$ and the average kinetic energy ${\bar{E_{k}}}_{i}$ were computed. Recall that *i* typically ranges from 1 to 500. For a given participant in a given condition, we computed $E_{km}=E\left(\bar{E_{ki}}\right)$, $f_{m}=E\left(f_{i}\right)$, and $I=\frac{E_{km}}{\pi \;f_{m}}$, where $E(x_{i})$ denotes the arithmetic mean of an arbitrary set of values $x_{i}$. Equation (1) is valid for all values of $\left(f_{i},{\bar{E_{k}}}_{i}\right)$, and we assume that *I* is constant according to adiabatic invariant theory [14]. Taking into account Equation (1) together with $E_{km}=\pi \;I\;f_{m}$ leads to

(2) $$\frac{\bar{E_{ki}}}{E_{km}}=\frac{f_{i}}{f_{m}}.$$

Therefore, a prediction of our model is that $\frac{\bar{E_{ki}}}{E_{km}}$ versus $\frac{f_{i}}{f_{m}}$ should behave as a straight line with slope 1 and a zero intercept.

After each gait cycle was identified, an average cycle was computed. To do this, each cycle was normalized to a unit duration and a spline of 1200 equally spaced points was computed for each cycle. Then, 1200 bins—one for each frame—were created and filled with the data from the splines of all cycles of a given participant in a given condition. The mean and standard deviation were computed for each bin.

### 2.3. SI Variability, Predictability, and Complexity

**Figure 2:**
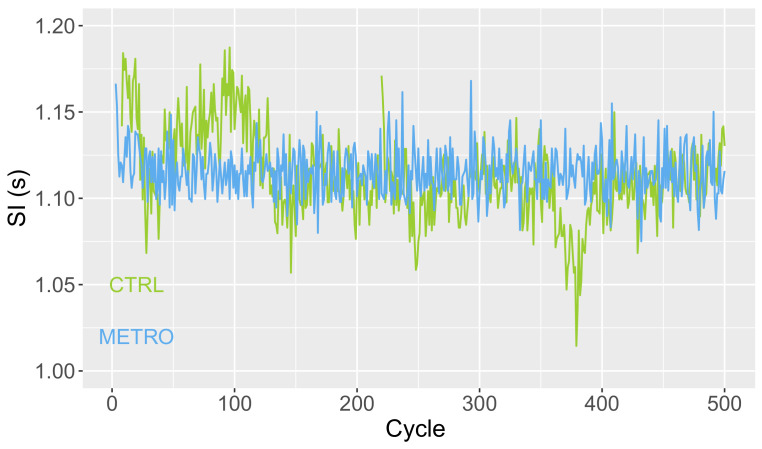
Typical plots of the SI time series obtained in the two studied conditions: CTRL (green) and METRO (blue). Parameters in the CTRL condition are: SI = 1.12 s, CV = 0.0282, H = 0.988, S = 1.59, and D = 1.35. Parameters in METRO condition are: SI = 1.12 s, CV = 0.0132, H = 0.383, S = 2.20, and D = 1.51.

Time series T with durations $T_{i}$ of successive gait cycles were computed from heel strikes of the right foot identified by CueFors 2 software. At the end of the session, two time series were generated for each subject in the CTRL and METRO conditions. Typical plots are shown in Figure 2.

First, the average SI was computed, as well as the coefficient of variation, CV = SD(T)/SI, estimating the magnitude of SI fluctuations. The Hurst exponent, H, was then computed by resorting to Detrended Fluctuation Analysis following the guidelines in [6]; more technical details about the algorithm we used can be found in [24]. By definition, *H* is mainly a measure of the time series’ predictability [4,5]. Therefore, it is relevant to complement it with other variability indices [8,9,25], of which we have chosen the Minkowski fractal dimension, D, [24], and the sample entropy, S, both as measures of complexity [1]. Computational details for D can be found in [24], while S was computed using the method described in [26]. All SI data analysis was performed with R (v. 4.1.0).

Figure 2 gives a first hint of SI variability, predictability, and complexity. Fluctuations around the mean value have a smaller magnitude in the METRO condition (smaller CV), but a simpler temporal structure. Indeed, the latter fluctuations show increasing or decreasing trends during several tenths of cycles in CTRL conditions, resulting in a larger predictability (larger H). The lower predictability in the METRO condition also results in a larger sample entropy, meaning that the fluctuations are closer to a random process in the METRO condition. Indeed, S is maximal for a random process.

As discussed in Appendix A, CV provides an estimate of ϵ, the magnitude of the time-dependent noise modeling the quasiperiodic nature of human motion. As our approach is only valid for ϵ well smaller than 1, the measured values of CV must be well smaller than 1 too, for our approach to be consistent.

### 2.4. Statistical Analysis

Data assessing SI variability, predictability, and complexity were tested for normality (Shapiro–Wilk) and equality of variance test. A paired *t*-test was performed and used to examine the effects of condition (CTRL or METRO) on SI, CV, H, D, and S. The significance level was set at p=0.05. In the case of a failed normality test, a Wilcoxon signed rank test was performed. The adiabatic invariant *I* was compared in the CTRL and METRO conditions using the same methodology. All these statistical procedures were performed with SigmaPlot software version 11.0 (Systat Software, San Jose, CA, USA).

An ANCOVA with zero intercept and significance level p=0.05 was performed to compare the linear trends of $\frac{\bar{E_{k}}}{E_{km}}$ versus $\frac{f}{f_{m}}$ in conditions CTRL and METRO, i.e., according to model

(3) $$\frac{\bar{E_{k}}}{E_{km}}=k\;\frac{f}{f_{m}},$$

where *k* is the experimentally observed slope. A linear regression with zero intercept of $\frac{\bar{E_{k}}}{E_{km}}$ versus $\frac{f}{f_{m}}$ was also performed independently of the condition, and the 95% confidence interval of the slope was computed. ANCOVA was performed with R software (v. 4.1.0).

Dynamic Time Warping (DTW), an algorithm developed to measure “distances” between similarly patterned time series, was then run to compare the distance between CTRL and METRO conditions for each participant whole-body COM vertical position (*Q*) and speed (*P*) as a function of time. The distances were computed for *Q* and compared to that computed for *P* using a paired *t*-test. The `dtw’ package in R was used, and the time series were z-normalized before comparison between the time series.

## 3. Results

### 3.1. Population

The general characteristics of our participants can be found in Table 1.

### 3.2. SI Variability, Predictability, and Complexity

**Figure 3:**
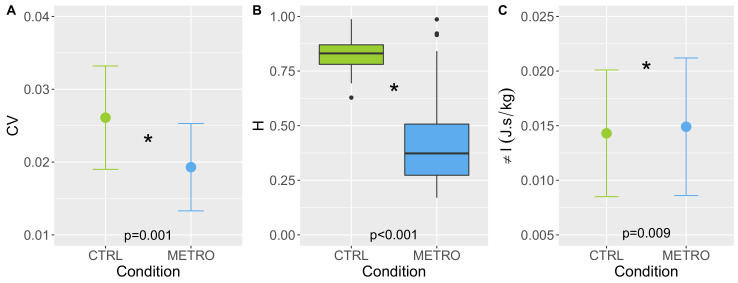
(**A**) Comparison of the mean values of CV in CTRL and METRO conditions. The error bar is equal to 1 SD. (**B**) Boxplots comparing the distribution of H in CTRL and METRO conditions. (**C**) Same graphical representation as in (**A**) for πI. The stars (*) denote significant differences between the means or medians between the two conditions.

A comparison of the results obtained in the CTRL and METRO conditions in the analysis of the SI time series is shown in Table 2. SI and D are not significantly changed. CV and H significantly decreased in the METRO condition, with H > 0.5 in the CTRL condition and <0.5 in the METRO condition. S marginally increased in the METRO condition. The significant differences are shown in Figure 3 graphically.

### 3.3. Phase-Space Dynamics

The adiabatic invariant is significantly increased in METRO condition: πI = 0.0149 ± 0.0063 J·s/kg against πI = 0.0143 ± 0.0058 J·s/kg (p=0.009). The difference is displayed in Figure 3.

**Figure 4:**
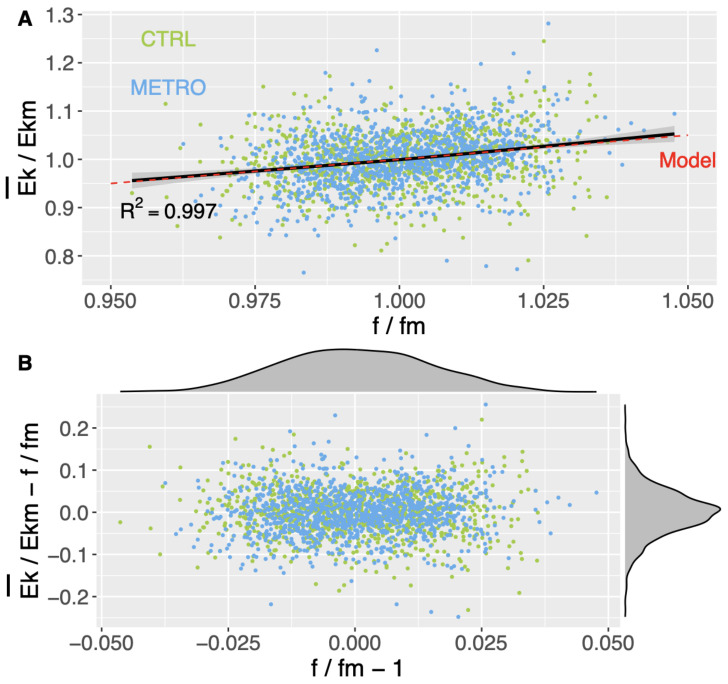
(**A**) Computed pairs $\left(\frac{f}{f_{m}},\frac{\bar{E_{k}}}{E_{km}}\right)$ (points) compared to a global regression of the form (Equation (3)) (black line and gray band indicating the 95% confidence interval). The coefficient of determination $R^{2}$ of the regression is indicated. The model (Equation (2)) is also shown (dashed red line). (**B**) Computed pairs with the linear trend (2) removed $\left(\frac{f}{f_{m}}-1,\frac{\bar{E_{k}}}{E_{km}}-\frac{f}{f_{m}}\right)$. The densities of the points along the two axes are shown above (density of $\frac{f}{f_{m}}$) and to the right side of the plot (density of $\frac{\bar{E_{k}}}{E_{km}}-\frac{f}{f_{m}}$).

The linear trend (Equation (3)) is confirmed by the ANCOVA (p<0.001), and the slope does not depend on the condition (p=0.700). The observed slope has a 95% confidence interval of [0.998,1.002]: k=1 is compatible with the latter interval. The quality of the linear regression can be graphically appraised in Figure 4A. Figure 4B shows the dispersion of the data around the linear relation (2). It can be seen that the values of $\frac{\bar{E_{k}}}{E_{km}}-\frac{f}{f_{m}}$ are well peaked around the zero value, which is related to the high value reached for $R^{2}$.

The average DTW distance between the CTRL and METRO conditions was 10.3±3.8 for *Q* and 12.9±7.4 for *P*. A paired *t*-test finds these values not statistically different with p=0.099.

## 4. Discussion

The objectives of this study were twofold: (1) to investigate whether the adiabatic invariant model (Equation (1)) holds in a population of healthy young adults walking at spontaneous speed on a motorized treadmill, and (2) to understand how a constraint applies on the system with a metronome impact global stability and SI variables. The major findings were that: (1) the invariant model was verified, and (2) SI variabilty (CV) and predictability (H) significantly decreased in the METRO condition, and global stability (*I*) significantly increased in the METRO condition.

The originality of this work is that it simultaneously measured the global stability, and local variability, predictability, and complexity of motorized-treadmill walking in two conditions. In the first, CTRL, participants walked at spontaneous speed. In the second, METRO, participants adjusted heel strikes to a tone emitted by a metronome whose frequency matched each participant’s preferred frequency. The latter condition is known to induce significant changes in step-to-step variation [11], and this was also the case in our study. We first comment on the SI variability, predictability, and complexity results. The values we found are typical of the long-range variability observed in healthy young adults; see, e.g., [9,24] for CV, H, and D, and [25] for S. A salient feature of our results is the decrease in H from correlated (H = 0.848) to anti-correlated (H = 0.373) values. This phenomenon was previously observed in [11]. The metronome that clicks at the spontaneous frequency of a participant “destroys” the normal SI autocorrelation pattern [11]. This can be interpreted by the additional constraint that the metronome imposes. Here, the participant is not free to adapt his/her variability, as would be the case with an optimal motor strategy, but must inhibit any change in SI variability to remain synchronous with the metronome. This blocking mechanism leads to anti-correlation, and also to a much smaller CV in the METRO condition. Note that CV is much smaller than 1 in both conditions, and so it is consistent with our mechanical approach, where the magnitude of the time-dependent noise is assumed to be small. S is almost significantly higher in the METRO condition. This could be related to the fact that the q3 value of H in METRO condition is equal to 0.56: a non-negligible fraction of our participants has H around 0.5, and random variability has maximum entropy.

Our major observation is that the model (3) holds in both the CTRL and METRO conditions. Although the participants change their SI variability and predictability, the dynamical stability constraint induced by the adiabatic invariance is verified: $\bar{E_{k}}$ of the whole-body COM and *f* of a given cycle are linearly correlated. Note that the model (3) does not forbid changing the value of *I* in the different conditions. By the way, the adiabatic invariant *I* is significantly higher in the condition METRO. On the one hand, $I=\frac{\bar{E_{k}}}{\pi f}$. On the other hand, *f* does not change significantly in the METRO condition because *T* does not change. Thus, the increase in *I* is associated with a higher $\bar{E_{k}}$ per gait cycle. Regarding phase-space dynamics, the DTW distance for *P* is not significantly different from that for *Q*. This result does not allow us to infer that the change in *I* is mostly due to a change of behavior in one of the variables *P* or *Q*. Instead, it shows that the change in *I* is due to a simultaneous change of both *P* and *Q* behaviors.

Strategies for human locomotion based on adiabatic invariance have already been developed and computed from the total (translational and rotational) body’s kinetic energy per stride [19]. In this study, a multi-segmental model was used, in which the body segments were treated as an ensemble of systems in motion, each characterized within a stride by the summed changes in kinetic energies from and about their respective centers-of-mass. In this model, walking is viewed as a sequence of joint rotations, and is referred to as a “segmental” approach [27]. Here, we extend the results of this previous work, as we were able to show the validity of the theory of adiabatic invariance in walking using a single-point kinematic model represented by the vertical component of the whole-body COM. The latter is known to provide summarized information about all body segments during walking. In contrast to the previous model, walking is considered as achieving a forward motion of the whole-body system [27]. Even more interesting is that the vertical displacement of the whole-body COM is related to the metabolic cost [28]. Therefore, we hypothesize that *I* is related to the metabolic cost of walking, and that participants are able to follow the metronome constraint at the expense of a larger oxygen consumption ($\dot{V}O_{2}$). This picture is coherent with [29], in which it is shown that an altered SI variability correlates with higher $\dot{V}O_{2}$. However, the changes in variability in the latter study were due to different walking speeds, which are not comparable with our protocol. It has also been shown that the nervous system uses predictions of the optimal gait to optimize the energetic cost of each new step [30]. This has also been demonstrated in other activities such as pedalling [31]. We think that keeping a constant value for *I* during walk with a given set of external conditions is one of the constraints involved in the prediction of the nervous system. The presence of such a constraint may actually improve the efficiency of prediction by excluding irrelevant motor strategies and reducing the degrees of freedom of the neuro-musculoskeletal system [22].

From a methodological viewpoint, we choose to compute $\bar{E_{k}}$ of the whole-body COM only in the vertical direction (sagittal plane motion), and not in the anteroposterior and mediolateral directions (horizontal and transverse plane motion). This choice could be justified by the methodology implemented for SI variables assessment requiring numerous gait cycles. Walking on a motorized treadmill is not similar to walking on the ground, and station-keeping on the belt is impossible and surely less important than not falling [32]. Displacement of the whole-body COM in the horizontal and transverse planes is mainly related to foot placement dynamics on the belt that may be affected to the motion strategy adopted by the participants; for example, if they want to avoid the belt’s edge [32]. Therefore, the displacement of the COM in horizontal and transverse planes was excluded from our analyses, since the motion in this plane is not representative of a spontaneous motion from a walk on the ground. Moreover, a drift correction method should have been considered before calculating the adiabatic invariants, which is out of the scope of our paper. As a consequence, only one adiabatic invariant has been computed, while from the separability hypothesis, three adiabatic invariants should be possibly computed from the COM motion during walk, one per direction. Further studies are now necessary to explore this statement.

The present study has some limitations that should be addressed. First, we considered the CTRL condition as a reference to compare the effects of the metronome. However, walking on a motorized treadmill results in an anti-correlated pattern in the stride speed fluctuations [11]. Therefore, the best reference condition is walking on the ground, but recording the kinematics of the pelvis during a large number of cycles is not possible without moving to half-turns in the calibrated volume, which would lead to a disturbance of the locomotor rhythm. Second, $\dot{V}O_{2}$ was not measured during walking. However, a direct relationship between total body kinetic energy and $\dot{V}O_{2}$ was previously observed at various constant walking speeds [19]. The measurement of $\dot{V}O_{2}$ would have allowed us to test our hypothesis formulated above regarding the relationship between *I* and the metabolic cost of walking. Third, we use a reduced kinematic pelvic model with four markers to estimate the whole-body COM position instead of a whole-body kinematic model. However, we believe that our pelvic model is more robust than the single sacral marker method, which is a too rough approximation of COM, given that the latter can move with respect to the sacrum [27]. The pelvic model is also theoretically controlled for pelvic tilt motion [33], and assumes that the pelvis position could be an approximation of the whole-body COM position [32]. More, the pelvic model is favored by clinicians during routine gait analysis to reduce the experimental and postprocessing times. Therefore, we hope that the reduced kinematic pelvic model used here will be an incentive to test the existence of global stability of the whole-body COM in pathological populations.

In summary, we have shown for the first time that an adiabatic invariant, *I* (see Equation (1)), is a robust dynamical stability constraint on SI variability and predictability. In other words, the vertical speeds and positions of one individual’s COM during successive walking cycles are not arbitrary, but such that *I* is invariant. The value of the adiabatic invariant does not change during walking, as expected from a mechanical model, although external perturbations (here, rhythmic auditory cues from a metronome + physiological noise) may change the latter value, arguably because a larger *I* is related to a larger energy expenditure in response to external perturbation. To what extent this constraint still holds in patients with motor disorders—e.g., Parkinson’s disease—and unveiling its relationship with physiological mechanisms are open problems that we hope to address in future work.

## Figures and Tables

**Table 1:** General characteristics of our population. Results are reported in the form mean ± SD. The number of gait cycles performed by the participants in 10 min is reported in the form median [q1–q3], regardless of the condition.

*N*	25
Age (years)	22.8 ± 5.2
Mass (kg)	68.1 ± 13.6
Height (m)	1.65 ± 0.32
Sex (M/F)	9/16
Gait cycles	532 [513–552]

M: male; F: female.

**Table 2:** Comparison between results in conditions CTRL and METRO for the SI analysis. Results are reported in the form mean±SD if a paired *t*-test was performed, or median and first-third quartiles [q1–q3] if a Wilcoxon signed rank test was performed. Significant *p*-values are in bold.

Condition	SI (s)	CV	H	D	S
CTRL	1.184 [1.126–1.269]	0.0261 ± 0.0071	0.848 [0.781–0.951]	1.633 ± 0.116	1.759 ± 0.228
METRO	1.187 [1.119–1.273]	0.0193 ± 0.0060	0.373 [0.265–0.560]	1.667 ± 0.129	1.891 ± 0.249
*p*	0.258	**0.001**	<**0.001**	0.282	0.063

SI: stride interval; CV: coefficient of variation; H: Hurst exponent; D: Minkowski fractal dimension; S: sample entropy.

## Data Availability

Data are available at https://osf.io/uxbdf accessed on 8 September 2022.

## Appendix A. Adiabatic Invariants and Random Noise

The dynamical state of a system coordinatized by configuration space variables $Q^{\alpha }$, α=1,…,n can be summarized by its trajectory in 2n-dimensional phase space $\left(Q^{\alpha },P_{\alpha }\right)$, where $P_{\alpha }$ are the momentum degrees of freedom. In standard dynamics, $P_{\alpha }=m\;{\dot{Q}}^{\alpha }$ with *m* a mass scale, although Hamiltonian dynamics can be formulated for more general systems. The trajectory is generated by a set of initial conditions $\left(Q_{0}^{\alpha },P_{0\alpha }\right)$ and an evolution operator called a Hamiltonian, $H(Q^{\alpha },P_{\alpha })$, which leads to the equations of motion (Ch. 45) [14].

(A1) $${\dot{Q}}^{\alpha }=\frac{\partial H}{\partial P_{\alpha }},\;{\dot{P}}_{\alpha }=-\frac{\partial H}{\partial Q_{\alpha }}.$$

If the Hamiltonian has a standard, separable, time-independent form, $H=\sum_{\beta =1}^{n}$$\left[\frac{1}{2}P_{\beta }^{2}+V_{\beta }\left(Q^{\beta }\right)\right]$, then ${\dot{Q}}^{\alpha }=P_{\alpha }$ and the momentum can be interpreted as the body velocity. Separability allows a focus on a Hamiltonian system with n=1, knowing that the results can be straightforwardly extended. Let us consider the Hamiltonian $H=\frac{P^{2}}{2}+V\left(Q\right)$. If the potential *V* allows for bounded trajectories in phase space, the action variable can be defined as

(A2) $$I=\frac{1}{2\pi }\oint_{\Gamma }P\;dQ\;,$$

where Γ is the bounded trajectory in the plane (Q,P). The relation (Equation (1)) is equivalent to (Equation (A2)) in our case. Then, the Hamiltonian can be reformulated as a function of only the action variables: $H=H_{0}\left(I\right)\;$, which is always true for an integrable system. The equations of motion for the integrable system now reads (Ch. 45) [14]

(A3) $$\dot{I}=-\frac{\partial H_{0}}{\partial \theta }=0,\;\dot{\theta }=\frac{\partial H_{0}}{\partial I}=\omega \left(I\right),$$

where θ is the angle variable. The action variables are therefore invariant and $\theta =\omega \left(I\right)\;t+\theta_{0}$.

In attempting to model the quasiperiodic nature of human motion, it is of interest to assume that

(A4) $$H=H_{0}\left(I\right)+\epsilon \;\xi \left(t\right)\;V\left(I,\theta \right),$$

where 0<ϵ≪1, and where ξ(t) is a stochastic noise with vanishing mean value. It is also assumed that $H_{0}\left(I\right)$ must satisfy the assumptions underlying the Nekhoroshev theorem [34,35]. According to (theorems 1.1 and 3.1) [36], the action variable becomes time-dependent because of the random noise (*I* becomes $I_{\epsilon }\left(t\right)$), but the deviation from the value *I* (which can be taken as the initial value) remains small, of order $\sqrt{\epsilon }\;$, up to a time of order 1/ϵ or even better [37], up to time of order $1/\epsilon^{2}\;$ . More precisely, $|I_{\epsilon }\left(t\right)-I|=\sqrt{\epsilon }\;Y\left(t\right)$ with Y(t) being a Gaussian Markov process.

Assuming that deviations from the time-independent dynamics are of order ϵ, a given observable Λ should behave approximately as Λ(t)=Λ(1+ϵf(t)). Hence, the coefficient of variation of Λ, $CV_{\Lambda }$, defined as the ratio between its standard deviation and its mean value Λ, is of order ϵ: $CV_{\Lambda }∼\epsilon$.

## References

[1] van Emmerik R.E., Ducharme S.W., Amado A.C., Hamill J. (2016). Comparing dynamical systems concepts and techniques for biomechanical analysis. J. Sport Health Sci..

[2] Hausdorff J., Peng C., Ladin Z., Wei J., Goldberger A. (1995). Is walking a random walk? Evidence for long-range correlations in stride interval of human gait. J. Appl. Physiol..

[3] Hausdorff J., Mitchell S., Firtion R., Peng C., Cudkowicz M., Wei J., Goldberger A. (1997). Altered fractal dynamics of gait: Reduced stride-interval correlations with aging and Huntington’s disease. J. Appl. Physiol..

[4] Hurst H.E. (1951). Long-Term Storage of Reservoirs: An Experimental Study. Trans. Am. Soc. Civ. Eng..

[5] Kantz H., Schreiber T. (1997). Nonlinear Time Series Analysis.

[6] Ravi D.K., Marmelat V., Taylor W.R., Newell K.M., Stergiou N., Singh N.B. (2020). Assessing the Temporal Organization of Walking Variability: A Systematic Review and Consensus Guidelines on Detrended Fluctuation Analysis. Front. Physiol..

[7] Moon Y., Sung J., An R., Hernandez M.E., Sosnoff J.J. (2016). Gait variability in people with neurological disorders: A systematic review and meta-analysis. Hum. Mov. Sci..

[8] Dierick F., Vandevoorde C., Chantraine F., White O., Buisseret F. (2021). Benefits of nonlinear analysis indices of walking stride interval in the evaluation of neurodegenerative diseases. Hum. Mov. Sci..

[9] Phinyomark A., Larracy R., Scheme E. (2020). Fractal Analysis of Human Gait Variability via Stride Interval Time Series. Front. Physiol..

[10] Dierick F., Buisseret F., Renson M., Luta A.M. (2020). Digital natives and dual task: Handling it but not immune against cognitive-locomotor interferences. PLoS ONE.

[11] Terrier P., Dériaz O. (2012). Persistent and anti-persistent pattern in stride-to-stride variability of treadmill walking: Influence of rhythmic auditory cueing. Hum. Mov. Sci..

[12] Ahn J., Hogan N. (2013). Long-Range Correlations in Stride Intervals May Emerge from Non-Chaotic Walking Dynamics. PLoS ONE.

[13] Gates D., Su J., Dingwell J. (2007). Possible biomechanical origins of the long-range correlations in stride intervals of walking. Phys. A Stat. Mech. Its Appl..

[14] Landau L., Lifchitz E. (1988). Physique Théorique Tome 1: Mécanique.

[15] Jose J., Saletan E. (1998). Classical Dynamics: A Contemporary Approach.

[16] Boulanger N., Buisseret F., Dehouck V., Dierick F., White O. (2021). Motor strategies and adiabatic invariants: The case of rhythmic motion in parabolic flights. Phys. Rev. E.

[17] Kugler P., Turvey M., Schmidt R., Rosenblum L. (1990). Investigating a Nonconservative Invariant of Motion in Coordinated Rhythmic Movements. Ecol. Psychol..

[18] Kadar E., Schmidt R., Turvey M. (1993). Constants underlying frequency changes in biological rhythmic movements. Biol. Cybern..

[19] Turvey M., Holt K., Obusek J., Salo A., Kugler P.N. (1996). Adiabatic transformability hypothesis of human locomotion. Biol. Cybern..

[20] Broscheid K.C., Dettmers C., Vieten M. (2018). Is the Limit-Cycle-Attractor an (almost) invariable characteristic in human walking?. Gait Posture.

[21] Raffalt P., Kent J., Wurdeman S.R., Stergiou N. (2020). To walk or to run—A question of movement attractor stability. J. Exp. Biol..

[22] Bernstein N. (1967). The Co-Ordination and Regulation of Movements.

[23] R Core Team (2021). R: A Language and Environment for Statistical Computing.

[24] Dierick F., Nivard A., White O., Buisseret F. (2017). Fractal analyses reveal independent complexity and predictability of gait. PLoS ONE.

[25] Crevecoeur F., Bollens B., Detrembleur C., Lejeune T. (2010). Towards a “gold-standard” approach to address the presence of long-range auto-correlation in physiological time series. J. Neurosci. Methods.

[26] Yentes J., Hunt N., Schmid K., Kaipust J., McGrath D., Stergiou N. (2013). The Appropriate Use of Approximate Entropy and Sample Entropy with Short Data Sets. Ann. Biomed. Eng..

[27] Tesio L., Rota V. (2019). The Motion of Body Center of Mass During Walking: A Review Oriented to Clinical Applications. Front. Neurol..

[28] Ortega J.D., Farley C.T. (2005). Minimizing center of mass vertical movement increases metabolic cost in walking. J. Appl. Physiol..

[29] Rock C.G., Marmelat V., Yentes J.M., Siu K.C., Takahashi K.Z. (2018). Interaction between step-to-step variability and metabolic cost of transport during human walking. J. Exp. Biol..

[30] Selinger J.C., Wong J.D., Simha S.N., Donelan J.M. (2019). How humans initiate energy optimization and converge on their optimal gaits. J. Exp. Biol..

[31] Takaishi T., Yasuda Y., Moritani T. (1994). Neuromuscular fatigue during prolonged pedalling exercise at different pedalling rates. Eur. J. Appl. Physiol. Occup. Physiol..

[32] Wang Y., Srinivasan M. (2014). Stepping in the direction of the fall: The next foot placement can be predicted from current upper body state in steady-state walking. Biol. Lett..

[33] Saini M., Kerrigan D.C., Thirunarayan M.A., Duff-Raffaele M. (1998). The Vertical Displacement of the Center of Mass During Walking: A Comparison of Four Measurement Methods. J. Biomech. Eng..

[34] Nekhoroshev N. (1971). Behavior of Hamiltonian systems close to integrable. Funct. Anal. Its Appl..

[35] Nekhoroshev N. (1977). An exponential estimate of the time of stability of nearly-integrable Hamiltonian systems. Uspekhi Mat. Nauk..

[36] Khas’minskiĭ R.Z. (1966). On Stochastic Processes Defined by Differential Equations with a Small Parameter. Theory Probab. Its Appl..

[37] Cogburn R., Ellison J.A. (1992). A stochastic theory of adiabatic invariance. Commun. Math. Phys..

